# Crystal structure of 2-(4-methyl­benzyl­idene)malono­nitrile

**DOI:** 10.1107/S1600536814024660

**Published:** 2014-11-15

**Authors:** Ouafa Amiri, El Mostapha Rakib, Abdellah Hannioui, Mohamed Saadi, Lahcen El Ammari

**Affiliations:** a Laboratoire de Chimie Organique et Analytique, Université Sultan Moulay Slimane, Faculté des Sciences et Techniques, Béni-Mellal, BP 523, Morocco; b Laboratoire de Chimie du Solide Appliquée, Faculté des Sciences, Université Mohammed V-Agdal, Avenue Ibn Battouta, BP 1014, Rabat, Morocco

**Keywords:** crystal structure, benzyl­idene, malono­nitrile, tyrphostins, benzyl­idenemalono­nitrile derivatives

## Abstract

The mol­ecule of the title compound, C_11_H_8_N_2_, is approximately planar (r.m.s.deviation for all non-H atoms = 0.023 Å). The malono­nitrile C—C—C angle is 113.54 (13)°. In the crystal, mol­ecules stack head-to-tail along [010]. There are no significant inter­molecular inter­actions present.

## Related literature   

For the pharmacological activity of benzyl­idenemalono­nitriles, see: Gazit *et al.* (1989 ▶); Levitzki & Mishani (2006 ▶). For the use of benzyl­idenemalono­nitrile derivatives in the preparation of heterocyclic compounds, see: Kolla & Lee (2011 ▶); Gao & Du (2012 ▶); Li *et al.* (2006 ▶).
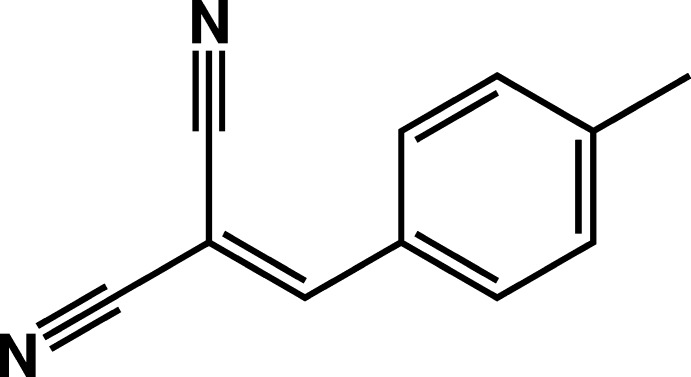


## Experimental   

### Crystal data  

C_11_H_8_N_2_
*M* *_r_* = 168.19
Triclinic, 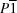

*a* = 7.0043 (5) Å
*b* = 7.5270 (5) Å
*c* = 9.5396 (6) Å
α = 106.757 (4)°
β = 96.592 (4)°
γ = 105.204 (4)°
*V* = 454.75 (5) Å^3^
*Z* = 2
Mo *K*α radiation
μ = 0.08 mm^−1^
*T* = 296 K
0.40 × 0.34 × 0.30 mm

### Data collection  

Bruker X8 APEX diffractometer
Absorption correction: multi-scan (*SADABS*; Bruker, 2009 ▶) *T*_min_ = 0.637, *T*_max_ = 0.746
7629 measured reflections
1923 independent reflections
1535 reflections with *I* > 2σ(*I*)
*R*_int_ = 0.022

### Refinement  

*R*[*F* ^2^ > 2σ(*F* ^2^)] = 0.046
*wR*(*F* ^2^) = 0.132
*S* = 1.06
1923 reflections
118 parameters
H-atom parameters constrained
Δρ_max_ = 0.20 e Å^−3^
Δρ_min_ = −0.18 e Å^−3^

### 

Data collection: *APEX2* (Bruker, 2009 ▶); cell refinement: *SAINT* (Bruker, 2009 ▶); data reduction: *SAINT*; program(s) used to solve structure: *SHELXS97* (Sheldrick, 2008 ▶); program(s) used to refine structure: *SHELXL97* (Sheldrick, 2008 ▶); molecular graphics: *ORTEP-3 for Windows* (Farrugia, 2012 ▶); software used to prepare material for publication: *PLATON* (Spek, 2009 ▶) and *publCIF* (Westrip, 2010 ▶).

## Supplementary Material

Crystal structure: contains datablock(s) I. DOI: 10.1107/S1600536814024660/su5017sup1.cif

Structure factors: contains datablock(s) I. DOI: 10.1107/S1600536814024660/su5017Isup2.hkl

Supporting information file. DOI: 10.1107/S1600536814024660/su5017Isup3.cml

**Fig. 1.**
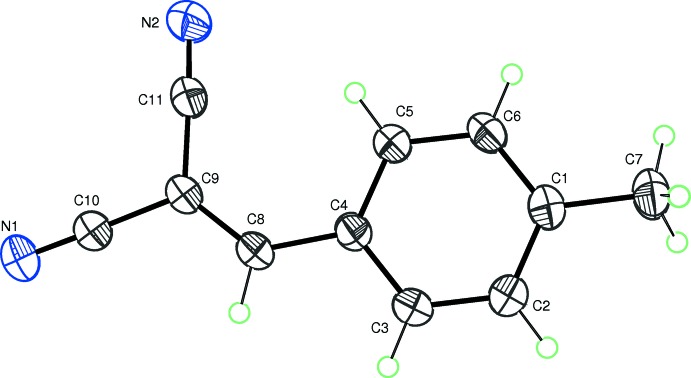
The molecular structure of the title compound, with atom labelling. Displacement ellipsoids are drawn at the 50% probability level.

. DOI: 10.1107/S1600536814024660/su5017fig1.tif
The mol­ecular structure of the title compound, with atom labelling. Displacement ellipsoids are drawn at the 50% probability level.

CCDC reference: 1033522

Additional supporting information:  crystallographic information; 3D view; checkCIF report

## supplementary crystallographic information

### S1. Comment

Pharmacological effects of benzylidenemalononitrile (BMN) compounds have been examined since the 1990's when several of their derivatives, referred to as tyrphostins, were recognized as specific inhibitors of epidermal growth factor tyrosine kinase (Gazit, *et al.*, 1989). Subsequent design and testing of a series of BMNs revealed new specific inhibitors of various protein tyrosine kinases (Levitzki & Mishani, 2006). It is well known that the benzylidenemalononitrile derivatives are very useful reagents for the preparation of heterocyclic compounds (Kolla & Lee, 2011; Gao & Du, 2012; Li *et al.*, 2006).

The title molecule is almost planar, Fig. 1, with an r.m.s. devation = 0.023 Å; the maximum deviation of -0.037 (2) Å was observed for atom C5. The malononitrile angle C10–C9–C11 is 113.58 (12)°.

In the crystal, molecules stack head-to-tail along [010]. There are no significant intermolecular interactions present.

### S2. Experimental

In a 250 ml round bottom flask, 4-methylbenzaldehyde (10 mmol), malononitrile (10 mmol) and phosphorus pentoxide (3.54 mmol) have stirred mechanically for ten minutes in 25 ml absolute ethanol. The resulting reaction mixture was heated at reflux using a water bath. The reaction mixture was poured onto crushed ice after the completion of the reaction monitored by TLC. On stirring separation of the desired product took place. The solid was filtered, washed with petroleum ether, dried and recrystallized from ethanol (yield: 68%, m.p.: 404 K), yielding block-like colourless crystals.

### S3. Refinement

H atoms were located in a difference Fourier map and treated as riding: C–H = 0.93 - 0.96 Å with *U*_iso_(H) = 1.5*U*_e_(C) for methyl H atoms and = 1.2*U*_eq_(C) for other H atoms.

### Crystal data

**Table:**

C_11_H_8_N_2_	*F*(000) = 176
*M**_r_* = 168.19	*D*_x_ = 1.229 Mg m^−^^3^
Triclinic, *P*1	Melting point: 404 K
*a* = 7.0043 (5) Å	Mo *K*α radiation, λ = 0.71073 Å
*b* = 7.5270 (5) Å	Cell parameters from 1923 reflections
*c* = 9.5396 (6) Å	θ = 3.0–27.1°
α = 106.757 (4)°	µ = 0.08 mm^−^^1^
β = 96.592 (4)°	*T* = 296 K
γ = 105.204 (4)°	Block, colourless
*V* = 454.75 (5) Å^3^	0.40 × 0.34 × 0.30 mm
*Z* = 2	

### Data collection

**Table:**

Bruker X8 APEX diffractometer	1923 independent reflections
Radiation source: fine-focus sealed tube	1535 reflections with *I* > 2σ(*I*)
Graphite monochromator	*R*_int_ = 0.022
φ and ω scans	θ_max_ = 27.1°, θ_min_ = 3.0°
Absorption correction: multi-scan (*SADABS*; Bruker, 2009)	*h* = −8→8
*T*_min_ = 0.637, *T*_max_ = 0.746	*k* = −9→9
7629 measured reflections	*l* = −12→12

### Refinement

**Table:**

Refinement on *F*^2^	Primary atom site location: structure-invariant direct methods
Least-squares matrix: full	Secondary atom site location: difference Fourier map
*R*[*F*^2^ > 2σ(*F*^2^)] = 0.046	Hydrogen site location: inferred from neighbouring sites
*wR*(*F*^2^) = 0.132	H-atom parameters constrained
*S* = 1.06	*w* = 1/[σ^2^(*F*_o_^2^) + (0.0567*P*)^2^ + 0.1308*P*] where *P* = (*F*_o_^2^ + 2*F*_c_^2^)/3
1923 reflections	(Δ/σ)_max_ < 0.001
118 parameters	Δρ_max_ = 0.20 e Å^−^^3^
0 restraints	Δρ_min_ = −0.18 e Å^−^^3^

### Special details

**Table:**

Geometry. All e.s.d.'s (except the e.s.d. in the dihedral angle between two l.s. planes) are estimated using the full covariance matrix. The cell e.s.d.'s are taken into account individually in the estimation of e.s.d.'s in distances, angles and torsion angles; correlations between e.s.d.'s in cell parameters are only used when they are defined by crystal symmetry. An approximate (isotropic) treatment of cell e.s.d.'s is used for estimating e.s.d.'s involving l.s. planes.
Refinement. Refinement of *F*^2^ against all reflections. The weighted *R*-factor *wR* and goodness of fit *S* are based on *F*^2^, conventional *R*-factors *R* are based on *F*, with *F* set to zero for negative *F*^2^. The threshold expression of *F*^2^ > σ(*F*^2^) is used only for calculating *R*-factors(gt) *etc*. and is not relevant to the choice of reflections for refinement. *R*-factors based on *F*^2^ are statistically about twice as large as those based on *F*, and *R*- factors based on all data will be even larger.

### Fractional atomic coordinates and isotropic or equivalent isotropic displacement parameters (Å^2^)

**Table:**

	*x*	*y*	*z*	*U*_iso_*/*U*_eq_	
C1	0.3051 (2)	−0.0064 (2)	0.22452 (16)	0.0427 (4)	
C2	0.1662 (3)	0.0403 (2)	0.30984 (17)	0.0488 (4)	
H2	0.0285	−0.0170	0.2695	0.059*	
C3	0.2306 (2)	0.1710 (2)	0.45410 (17)	0.0461 (4)	
H3	0.1352	0.2004	0.5095	0.055*	
C4	0.4357 (2)	0.25984 (19)	0.51829 (15)	0.0365 (3)	
C5	0.5753 (2)	0.2128 (2)	0.43179 (17)	0.0448 (4)	
H5	0.7131	0.2701	0.4714	0.054*	
C6	0.5089 (2)	0.0816 (2)	0.28791 (17)	0.0476 (4)	
H6	0.6036	0.0516	0.2321	0.057*	
C7	0.2353 (3)	−0.1472 (3)	0.06642 (17)	0.0555 (4)	
H7A	0.3430	−0.1963	0.0365	0.083*	
H7B	0.1977	−0.0811	0.0006	0.083*	
H7C	0.1209	−0.2539	0.0615	0.083*	
C8	0.4888 (2)	0.3945 (2)	0.67091 (15)	0.0389 (3)	
H8	0.3790	0.4133	0.7126	0.047*	
C9	0.6697 (2)	0.4962 (2)	0.76167 (15)	0.0386 (3)	
C10	0.6819 (2)	0.6205 (2)	0.91126 (16)	0.0435 (4)	
C11	0.8637 (2)	0.4944 (2)	0.72682 (17)	0.0476 (4)	
N1	0.6955 (2)	0.7184 (2)	1.03069 (15)	0.0601 (4)	
N2	1.0205 (2)	0.4968 (3)	0.70532 (18)	0.0734 (5)	

### Atomic displacement parameters (Å^2^)

**Table:**

	*U*^11^	*U*^22^	*U*^33^	*U*^12^	*U*^13^	*U*^23^
C1	0.0507 (9)	0.0391 (7)	0.0347 (7)	0.0136 (7)	0.0069 (7)	0.0079 (6)
C2	0.0390 (9)	0.0520 (9)	0.0436 (8)	0.0077 (7)	0.0051 (7)	0.0059 (7)
C3	0.0398 (9)	0.0523 (9)	0.0417 (8)	0.0131 (7)	0.0140 (7)	0.0080 (7)
C4	0.0388 (8)	0.0379 (7)	0.0320 (7)	0.0119 (6)	0.0091 (6)	0.0098 (6)
C5	0.0381 (9)	0.0524 (9)	0.0377 (8)	0.0135 (7)	0.0090 (6)	0.0061 (6)
C6	0.0467 (10)	0.0570 (9)	0.0368 (8)	0.0210 (8)	0.0132 (7)	0.0060 (7)
C7	0.0619 (12)	0.0541 (9)	0.0381 (8)	0.0160 (8)	0.0038 (8)	0.0012 (7)
C8	0.0406 (8)	0.0428 (7)	0.0331 (7)	0.0139 (6)	0.0128 (6)	0.0096 (6)
C9	0.0419 (9)	0.0405 (7)	0.0326 (7)	0.0146 (6)	0.0110 (6)	0.0083 (6)
C10	0.0412 (9)	0.0464 (8)	0.0384 (8)	0.0118 (7)	0.0102 (7)	0.0084 (6)
C11	0.0442 (10)	0.0522 (9)	0.0367 (8)	0.0146 (7)	0.0066 (7)	0.0014 (6)
N1	0.0602 (10)	0.0649 (9)	0.0403 (7)	0.0147 (7)	0.0136 (7)	−0.0016 (6)
N2	0.0460 (9)	0.0935 (13)	0.0613 (10)	0.0225 (9)	0.0134 (7)	−0.0043 (8)

### Geometric parameters (Å, º)

**Table:**

C1—C6	1.384 (2)	C6—H6	0.9300
C1—C2	1.389 (2)	C7—H7A	0.9600
C1—C7	1.508 (2)	C7—H7B	0.9600
C2—C3	1.382 (2)	C7—H7C	0.9600
C2—H2	0.9300	C8—C9	1.341 (2)
C3—C4	1.395 (2)	C8—H8	0.9300
C3—H3	0.9300	C9—C11	1.438 (2)
C4—C5	1.3998 (19)	C9—C10	1.4419 (18)
C4—C8	1.4535 (18)	C10—N1	1.1420 (19)
C5—C6	1.381 (2)	C11—N2	1.137 (2)
C5—H5	0.9300		
			
C6—C1—C2	118.20 (14)	C5—C6—H6	119.1
C6—C1—C7	121.04 (14)	C1—C6—H6	119.1
C2—C1—C7	120.75 (15)	C1—C7—H7A	109.5
C3—C2—C1	120.64 (15)	C1—C7—H7B	109.5
C3—C2—H2	119.7	H7A—C7—H7B	109.5
C1—C2—H2	119.7	C1—C7—H7C	109.5
C2—C3—C4	121.29 (14)	H7A—C7—H7C	109.5
C2—C3—H3	119.4	H7B—C7—H7C	109.5
C4—C3—H3	119.4	C9—C8—C4	130.79 (13)
C3—C4—C5	117.90 (13)	C9—C8—H8	114.6
C3—C4—C8	117.33 (12)	C4—C8—H8	114.6
C5—C4—C8	124.77 (14)	C8—C9—C11	126.43 (13)
C6—C5—C4	120.21 (15)	C8—C9—C10	120.02 (13)
C6—C5—H5	119.9	C11—C9—C10	113.54 (13)
C4—C5—H5	119.9	N1—C10—C9	178.50 (17)
C5—C6—C1	121.76 (14)	N2—C11—C9	177.20 (17)
