# Supplementary figures and images for: Crystal structure of 2-(4-methyl­benzyl­idene)malono­nitrile
Source: Acta Crystallogr Sect E Struct Rep Online. 2014 Nov 15;70(Pt 12):o1263. doi: 10.1107/S1600536814024660 (PMC4257432; doi:10.1107/S1600536814024660)

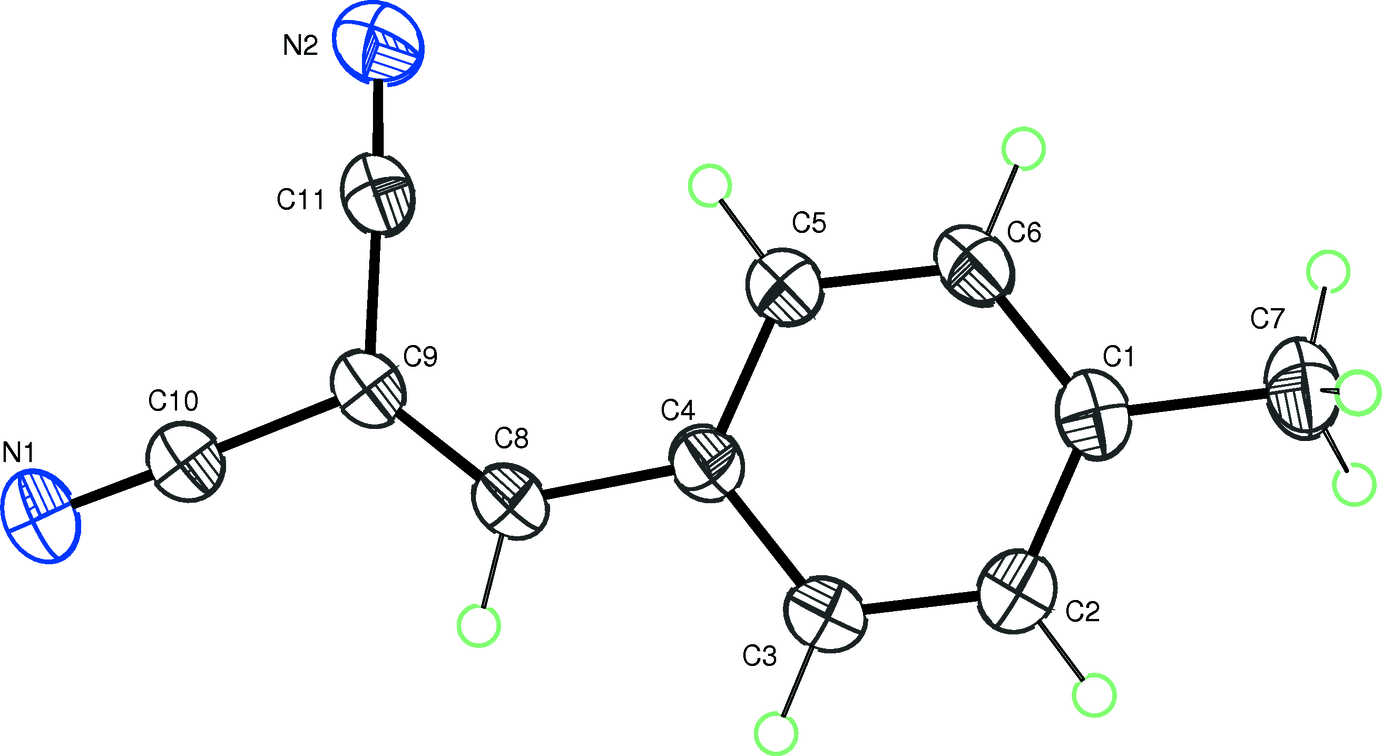

Supplement: Supplementary file 4 [file e-70-o1263-fig1.tif]
